# T-Cell Receptor CDR3 Loop Conformations in Solution Shift the Relative Vα-Vβ Domain Distributions

**DOI:** 10.3389/fimmu.2020.01440

**Published:** 2020-07-08

**Authors:** Monica L. Fernández-Quintero, Nancy D. Pomarici, Johannes R. Loeffler, Clarissa A. Seidler, Klaus R. Liedl

**Affiliations:** Center for Molecular Biosciences Innsbruck (CMBI), Department of General, Inorganic and Theoretical Chemistry, University of Innsbruck, Innsbruck, Austria

**Keywords:** CDR3 loop ensembles, conformational selection, Markov-state models, relative Vα/Vβ domain distributions, T-cell receptors, T-cell receptor structure and design

## Abstract

T-cell receptors are an important part in the adaptive immune system as they are responsible for detecting foreign proteins presented by the major histocompatibility complex (MHC). The affinity is predominantly determined by structure and sequence of the complementarity determining regions (CDRs), of which the CDR3 loops are responsible for peptide recognition. We present a kinetic classification of T-cell receptor CDR3 loops with different loop lengths into canonical and non-canonical solution structures. Using molecular dynamics simulations, we do not only sample available X-ray structures, but we also observe a substantially broader CDR3 loop ensemble with various distinct kinetic minima in solution. Our results strongly imply, that for given CDR3 loop sequences several canonical structures have to be considered to characterize the conformational diversity of these loops. Our suggested dominant solution structures could extend the repertoire of available canonical clusters by including kinetic minimum structures present in solution. Thus, the CDR3 loops need to be characterized as conformational ensembles in solution. Furthermore, the conformational changes of the CDR3 loops follow the paradigm of conformational selection, because the experimentally determined binding competent state is present within this ensemble of pre-existing conformations without the presence of the antigen. We also identify strong correlations between the CDR3 loops and include combined state descriptions. Additionally, we observe a strong dependency of the CDR3 loop conformations on the relative Vα-Vβ interdomain orientations, revealing that certain CDR3 loop states favor specific interface orientations.

**Graphical Abstract:**
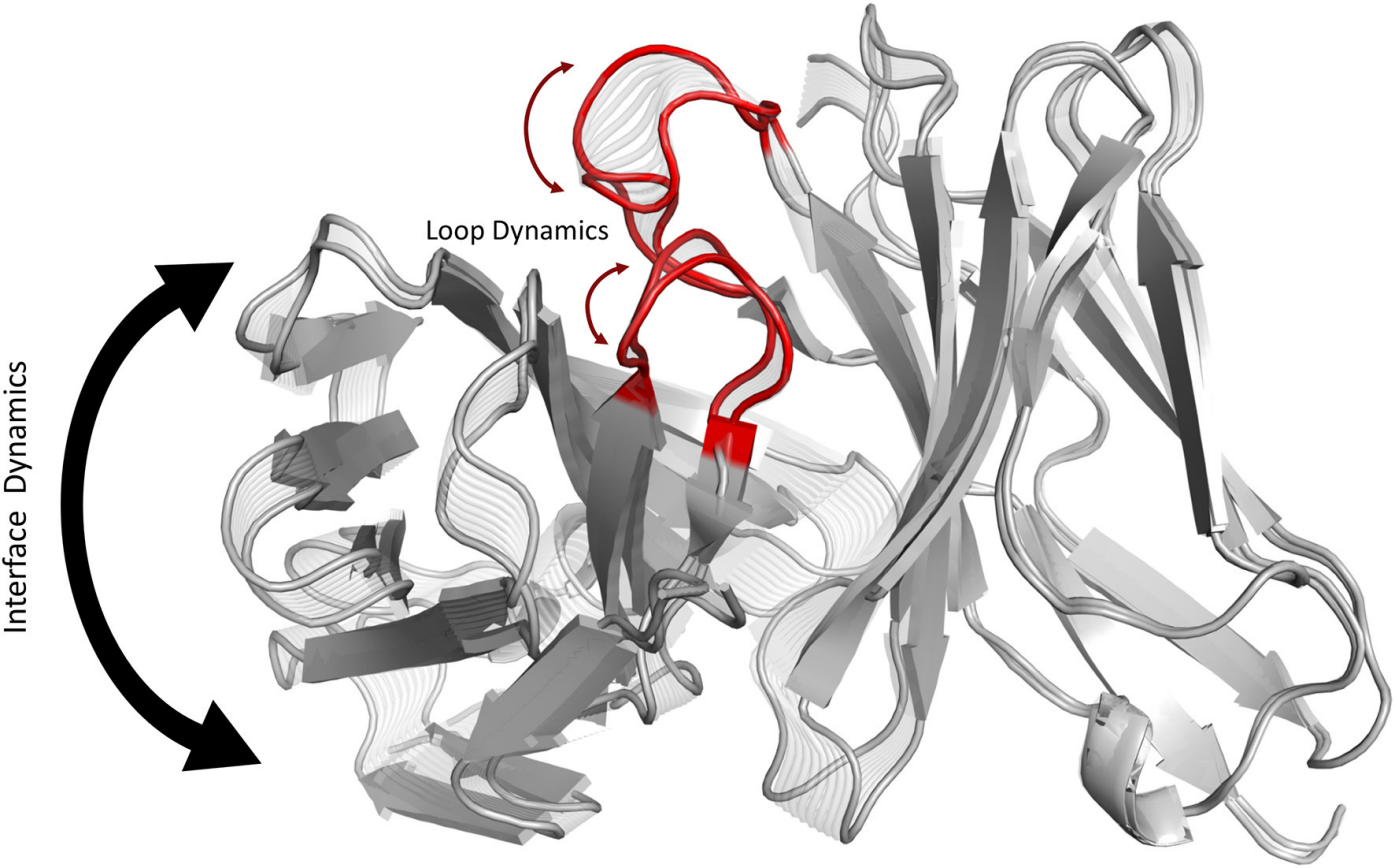
Graphical summary showing that conformational changes of the CDR3 loops can shift the relative Vα-Vβ interdomain orientations.

## Introduction

T-cell receptors (TCRs) play a fundamental role in the adaptive immune system and are responsible for recognizing foreign proteins (1). Depending on the type of the T-cell the TCR is expressed on, it recognizes protein fragments derived from intra- or extracellular regions, displayed by the Major Histocompatibility Complex (MHC) (2). TCRs consist of an α and a β chain analogous to the heavy and light chain in the antigen-binding fragment (Fab) of antibodies (3, 4). Vα and Cα can be interpreted as homologous in sequence and structure to the V_L_ and C_L_ domains in antibodies as well as Vβ and Cβ as equivalent to V_H_ and C_H_ (5, 6). The majority of T-cells are αβ T-cells, however, there also exist γδ T-cells, which are able to recognize a broad range of antigens without the presence of major histocompatibility complex molecules. γδ T-cells are an important subset of “unconventional” T lymphocytes, attacking target cells directly through their cytotoxic activity (7). TCR alpha chains are made by V and J genes, while beta chains are produced by V, D, and J genes. Sequence and structural diversity are concentrated on six hypervariable loops, also known as the complementarity determining regions (CDRs) (3, 8, 9). The CDR3α loop reveals a diversity of length and sequence composition due to the recombination of two gene segments V and J, while the CDR3β loop variability is increased due to the D gene (10, 11). The CDR3 loops are located in the center of the paratope and play a crucial role in the peptide recognition (12). Additionally, the CDR3 loops have been discussed to directly influence each other and to be structurally correlated (13, 14). Analogous to antibodies five of the six CDR loops adopt a limited set of main-chain conformations, so called canonical structures. Due to the high variability in sequence, length and the ability of the CDR3 loops to adopt different conformations during the V(D)J recombination, structure prediction remains challenging (15, 16). Compared to antibodies only few studies focused on characterizing and classifying TCR CDR loops into canonical classes (3, 9). Crystal structures of the same TCRs crystallized with and without pMHC (peptides presented by MHC molecules) reveal conformational changes upon binding mainly in the CDR3 loops (12, 17, 18). Compared to antibodies, TCRs reveal a higher flexibility in the CDR loops, especially in the CDR3 loops (16, 19). Analogous to CDR loops of antibodies, TCR CDR loops follow the concept of conformational diversity, because one TCR can adopt various different conformations, which directly influence the binding properties and their functions (9). The fact, that TCRs and antibodies are derived from a similar genetic mechanism and have a nearly identical architecture and a high structural similarity, inspires an antibody-like TCR design, TCR-mimic antibodies and soluble TCRs (5, 6, 10, 11, 20–22). Apart from the CDR loops, the binding site of TCRs is also known to be affected by the relative orientation of the variable domains, Vα and Vβ. The interdomain orientation influences the relative position of the CDR loops to one another and therefore changes the geometry of the antigen binding site (9, 23, 24). In addition to the variations in length and sequence of the CDRs, the modulation of the V_H_ and V_L_ interdomain orientation might not only be required means to accommodate the diverse antigenic shapes, but also a mechanism to increase the possible number of antibody paratopes (25). In this study, we investigate the conformational diversity of different TCR CDR3 loops and kinetically and thermodynamically classify the combined CDR3 loop ensembles in solution and their influence on the relative Vα-Vβ orientation. The investigated TCRs were chosen because of their strong experimental structural information and their differences in CDR3 loop lengths.

## Methods

Experimental structural information was available for all considered T-cell receptors. We deleted the co-crystallized antigen in all complex crystal structures. The starting structures for simulations were prepared in MOE (Molecular Operating Environment, Chemical Computing Group, version 2018.01) using the Protonate3D tool (26, 27). To neutralize the charges we used the uniform background charge (28–30). Using the tleap tool of the AmberTools18 (28, 29) package, the crystal structures were soaked with cubic water boxes of TIP3P water molecules with a minimum wall distance of 10 Å to the protein (31). For all crystal structures parameters of the AMBER force field 14SB were used (32). The TCRs were carefully equilibrated using a multistep equilibration protocol (33).

### Metadynamics Simulations

To enhance the sampling of the conformational space well-tempered metadynamics (34–36) simulations were performed in GROMACS (37, 38) with the PLUMED 2 implementation (39). We used metadynamics to overcome the timescale limitations of molecular dynamics simulations and to accelerate the sampling allowing the system to escape deep energy minima (35). As collective variables, we used a linear combination of sine and cosine of the ψ torsion angles of the CDR3α and CDR3ß loops, calculated with functions MATHEVAL and COMBINE implemented in PLUMED 2 (39). As discussed previously the ψ torsion angle captures conformational transitions comprehensively (40). The decision to include the CDR3α loop is based on the previously discussed structural correlation between the CDR3α and CDR3ß loops (13). The simulations were performed at 300 K in an NpT ensemble. We used a Gaussian height of 10.0 kcal/mol. Gaussian deposition occurred every 1,000 steps and a biasfactor of 10 was used. One micro second of metadynamics simulations were performed for each available TCR crystal structure. The constant and variable domains of each TCR were simulated (23). The resulting trajectories were clustered in cpptraj (29, 41) by using the average linkage hierarchical clustering algorithm with a distance cut-off criterion of 1.2 Å resulting in a large number of clusters. The cluster representatives for the TCR were equilibrated and simulated for 100 ns using the AMBER18 (42) simulation package.

### Molecular Dynamics Simulations

Molecular dynamics simulations were performed in an NpT ensemble using pmemd.cuda (43). Bonds involving hydrogen atoms were restrained by applying the SHAKE algorithm (44), allowing a time step of 2.0 fs. Atmospheric pressure of the system was preserved by weak coupling to an external bath using the Berendsen algorithm (45). The Langevin thermostat (46) was used to maintain the temperature during simulations at 300 K.

With the obtained trajectories we performed a time-lagged independent component analysis (tICA) using the python library PyEMMA 2 employing a lag time of 10 ns (47). Thermodynamics and kinetics were calculated with a Markov-state model (48) by using PyEMMA 2, which uses the k-means clustering algorithm (49) to define microstates and the PCCA+ clustering algorithm (50) to coarse grain the microstates to macrostates. The sampling efficiency and the reliability of the Markov-state model (e.g., defining optimal feature mappings) can be evaluated with the Chapman-Kolmogorov test (51, 52), by using the variational approach for Markov processes (53) and by taking into account the fraction of states used, as the network states must be fully connected to calculate probabilities of transitions and the relative equilibrium probabilities. The Chapman-Kolmogorov tests for all Markov-state models presented in this study are displayed in Figures S1–S5. To build the Markov-state model we used the backbone torsions of the respective CDR loop, defined 150 microstates using the k-means clustering algorithm and applied a lag time of 10 ns.

### Relative V_H_ and V_L_ Orientation Calculation

For the relative V_H_ and V_L_ orientations, described in this study, we defined a torsion angle between the center of mass (COM) of the CDR loops of the light chain, the COM of the V_L_ domain, the COM of the V_H_ domain and the COM of the CDR loops of the heavy chain.

## Results

**Figure 1:**
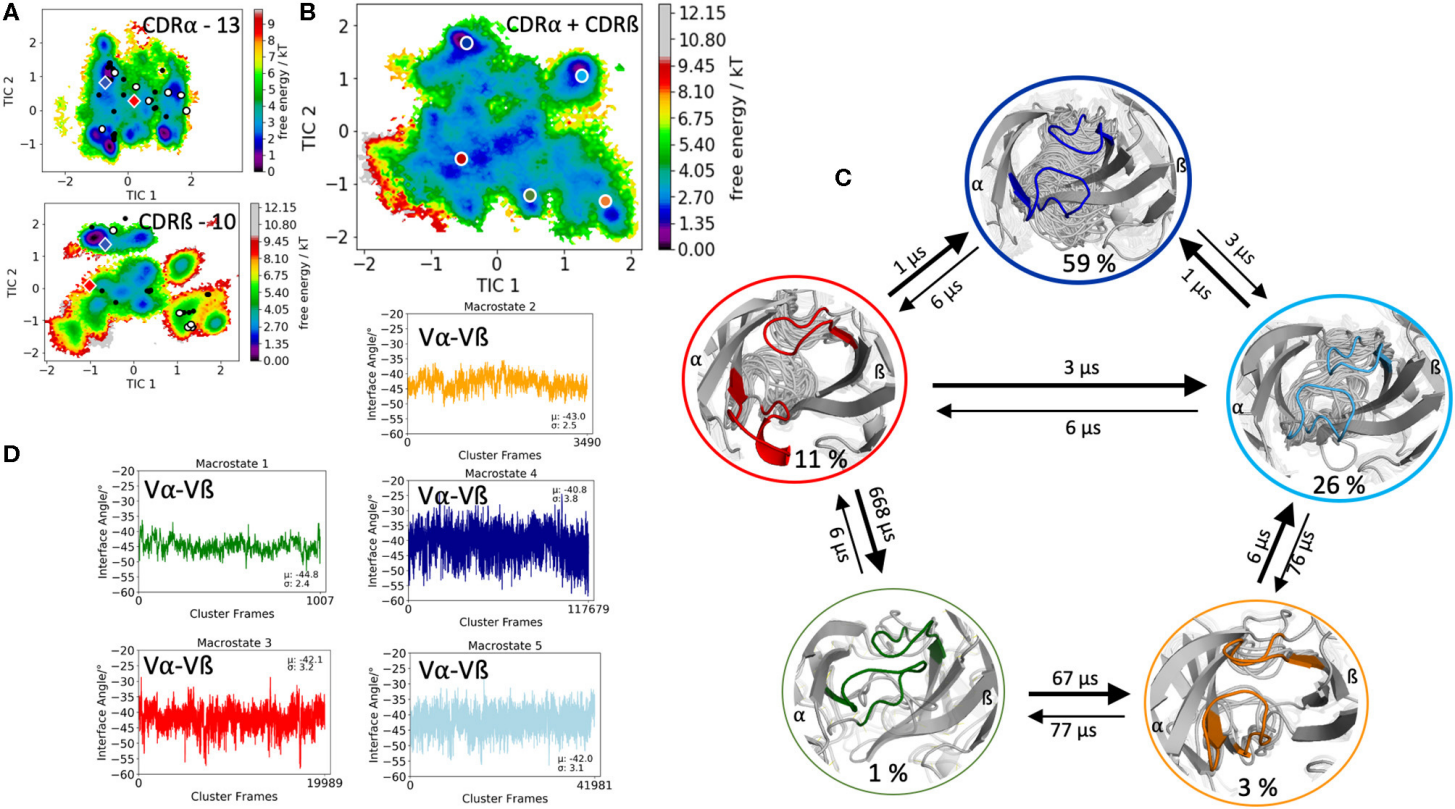
Free energy surfaces of the individual and combined CDR3 loops including a Markov-state model and the relative interdomain distributions upon conformational changes in the CDR3 loops of the B4.2.3 TCR. **(A)** Individual CDR3α and CDR3ß conformational spaces including the projections of the respective CDR3 loop crystal structures of same loop length. The X-ray structures crystallized without antigen, are colored in white, while the complexed structures are colored black. The respective CDR3 loop lengths are included in the individual free energy surfaces. Additionally, the crystal structures (diamond shapes) of the same TCR crystallized with (blue) and without (red) the pMHC complex are projected into the individual free energy landscapes. **(B)** Combined free energy surface of both CDR3 loops including the projections of the resulting five macrostate representatives. **(C)** Markov-state model, including the respective five macrostate ensembles and transition kinetics. The color-coding of the individual macrostates corresponds to the combined free energy surface in **(B)**. **(D)** Relative Vα-Vβ interdomain orientations for the individual macrostates shows significant shifts in the interface angle distributions for different CDR3 loop conformations. Again, the color-coding corresponds to **(B,C)**.

The first TCR studied, is the specific B4.2.3 TCR binding to the pMHC ligand P18-I10 H2-D^d^. Experimental structural information with and without the pMHC complex present (PDB accession codes: 5IVX, 5IW1) indicates large structural rearrangement of the CDR3 loops upon binding. As starting structure for 1 μs of metadynamics simulations we used the TCR structure crystallized in complex with the pMHC but simulated the TCR without the antigen. As described in the methods section, the resulting trajectory was clustered and the obtained 218 clusters, were used as starting structures for each 100 ns molecular dynamics simulations. The 21.8 μs of trajectories were then used to construct tICA free energy landscapes for the combined and the individual CDR3α and CDR3ß loops. The CDR3α loop length for this specific B4.2.3 TCR is 13, while the CDR3ß loop contains 10 amino acid residues. All available TCR crystal structures of the same CDR3 loop length with a resolution <2.2 Å were used for both the CDR3α and CDR3ß loops and projected into the respective individual free energy surfaces (Figure 1A). The majority of the available crystal structures are present within our obtained CDR3α and CDR3ß ensembles in solution, indicating that also the CDR3 loops in TCRs, analogous to antibodies, can adopt various different conformations in solution and thus need to be described as conformational ensemble. As it has been shown, CDR3α and CDR3ß loops are positively correlated and structurally influence each other, the combined conformational space of the CDR3 loops is shown in Figure 1B and the Markov-state model and transition times are shown in Figure 1C. We observe five macrostates and transition kinetics in the high microsecond timescale. Figure 1D displays the relative Vα-Vβ interdomain orientation of the respective CDR3 macrostate ensembles and we can clearly see a strong shift in the angle distributions upon conformational changes in the CDR3 loops.

**Figure 2:**
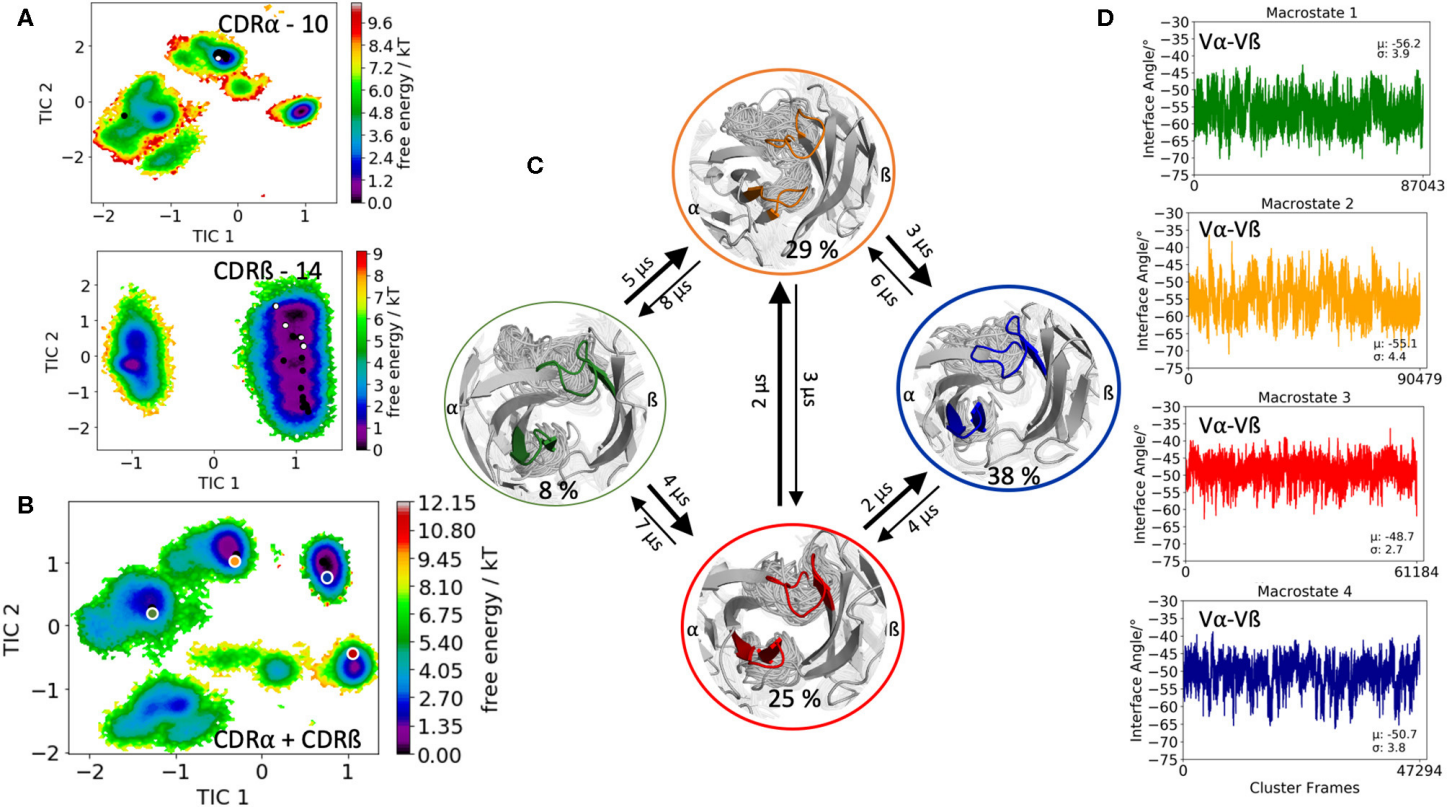
Free energy surfaces of the individual and combined CDR3 loops including a Markov-state model and the relative interdomain distributions upon conformational changes in the CDR3 loops of the mucosal-associated invariant TCR. **(A)** Individual CDR3α and CDR3ß conformational spaces including the projections of the respective CDR3 loop crystal structures of same loop length. The respective CDR3 loop lengths are included in the individual free energy surfaces. The X-ray structures crystallized without antigen, are colored in white, while the complexed structures are colored black. **(B)** Combined free energy surface of both CDR3 loops including the projections of the resulting four macrostate representatives. **(C)** Markov-state model, including the respective four macrostate ensembles and transition kinetics. The color-coding of the individual macrostates corresponds to the combined free energy surface in **(B)**. **(D)** Relative Vα-Vβ interdomain orientations for the individual macrostates exhibit significant shifts upon CDR3 loop rearrangements. Again, the color-coding corresponds to **(B,C)**.

The second TCR investigated, is the receptor of the mucosal-associated invariant T cells (MAIT cells) binding to the major-histocompatibility-complex-(MHC)-class-I-related molecule MR1 and a vitamin-B-based ligand (PDB accession code: 5U2V) (54). The CDR3α loop consists of 10 amino acid residues, while the CDR3ß contains 14 residues. Clustering of the 1 μs metadynamics trajectory resulted in 287 cluster representatives that were used as starting structures for each 100 ns of molecular dynamics simulations. Figure 2A shows the resulting conformational diversity of 28.7 μs of trajectories of the individual CDR3α and CDR3ß loops including the projection of the available TCR crystal structures of same length. We again clearly see that the majority of available TCR crystal structures for the CDR3α and CDR3ß loops lie within our ensemble in solution, stressing the fact that one single static structure is not enough to capture the high flexibility of these loops. Besides sampling the majority of crystal structures, Figure 2A shows additional dominant solution structures, which might be included when characterizing CDR3α and CDR3ß loop ensembles. The combined conformational space of the CDR3α and CDR3ß loops is illustrated in Figure 2B and reveals four macrostates with conformational transitions in the microsecond timescale (Figure 2C). Again, the obtained macrostate ensembles were used to calculate the relative Vα-Vβ interdomain distributions to identify the influence of different CDR3 loop conformations on the interface orientations. Excitingly, we observe substantial shifts in the Vα-Vβ interdomain distributions upon conformational changes in the CDR3 loops (Figure 2D).

**Figure 3:**
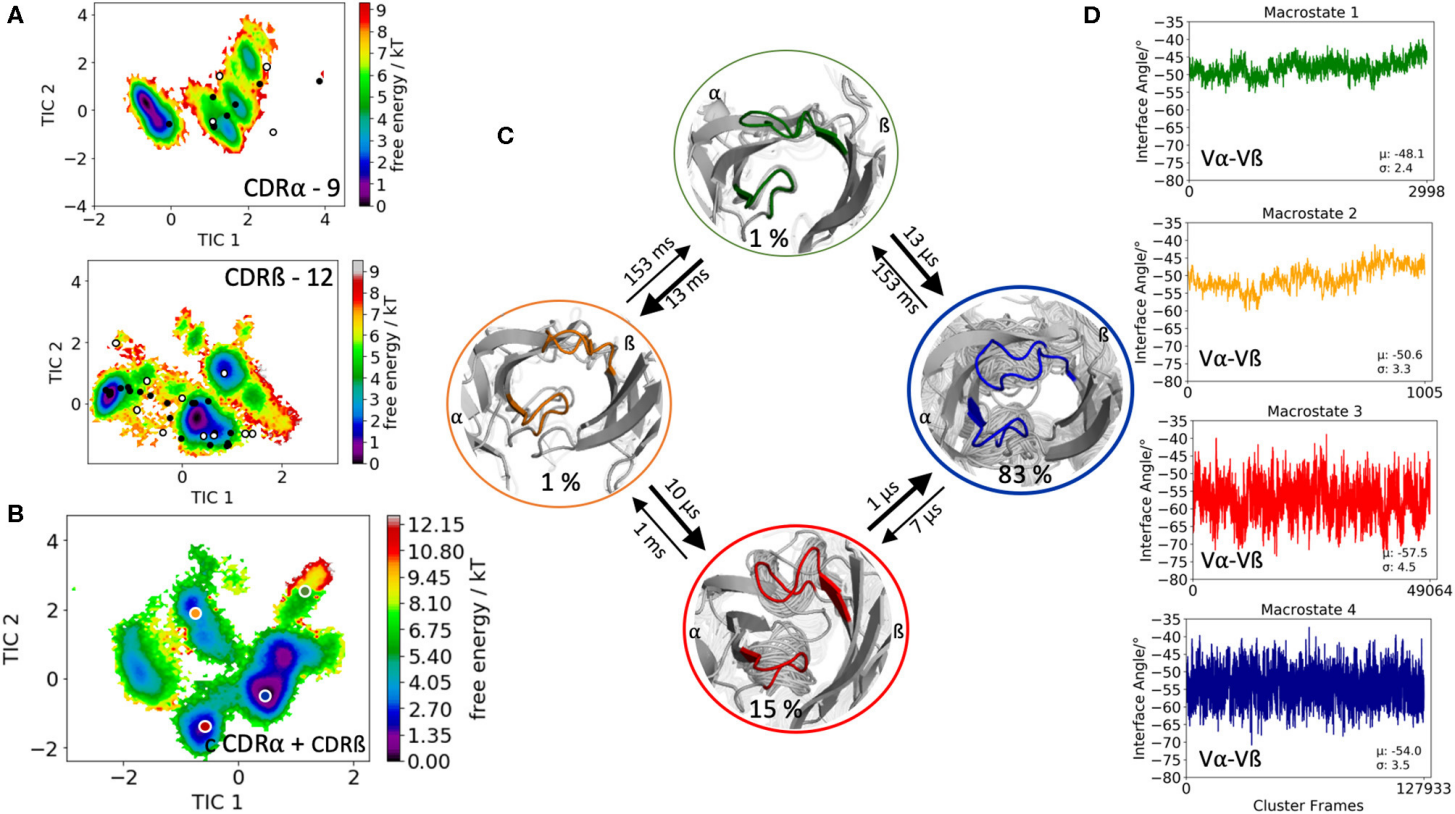
Free energy surfaces of the individual and combined CDR3 loops including a Markov-state model and the relative interdomain distributions upon conformational changes in the CDR3 loops of the 003 TCR recognizing HIV p17 Gag-derived peptide presented by HLA-A*0201. **(A)** Individual CDR3α and CDR3ß conformational spaces including the projections of the respective CDR3 loop crystal structures of same loop length. The respective CDR3 loop lengths are included in the individual free energy surfaces. The X-ray structures crystallized without antigen, are colored in white, while the complexed structures are colored black. **(B)** Combined free energy surface of both CDR3 loops including the projections of the resulting four macrostate representatives. **(C)** Markov-state model, including the respective four macrostate ensembles and transition kinetics. The color-coding of the individual macrostates corresponds to the combined free energy surface in **(B)**. **(D)** Relative Vα-Vβ interdomain orientations for the individual macrostates reveal substantial shifts upon conformational changes in the CDR3 loops. Again, the color-coding corresponds to **(B,C)**.

Another TCR studied, is part of an *in silico* and structural study investigating TCR loop flexibility and the influence of crystallization conditions and crystal packing effects on the loop flexibility (19). As starting structure for our simulation we used the 003 TCR that recognizes an HIV p17 Gag-derived peptide (SLYNTVATL) presented by HLA-A^*^0201 with the PDB accession code 6FR4. In this case the CDR3α loop length consists of 9 amino acids, while the CDR3ß loop is 12 residues long. The obtained 182 cluster representatives of the metadynamics simulations were used as starting structures for each 100 ns molecular dynamics simulations to reconstruct the free energy surfaces of the individual and the combined CDR3α and CDR3ß conformational spaces (Figures 3A,B). The results in Figure 3A clearly show the extraordinarily high flexibility of the individual CDR3 loops, in particular the CDR3ß loop, because the majority of available TCR crystal structures with the CDR3α loop length of 9 residues and the CDR3ß loop consisting of 12 residues are present within our obtained ensemble in solution. For the combined CDR3 loop states in Figure 3C we observe transition timescales in the micro-to-millisecond timescale. The macrostate representatives of the combined CDR3 loop tICA space are used to investigate the influence of different CDR3 loops on the relative interdomain distributions. Figure 3D illustrates the interface angle distributions of the respective macrostate ensembles and we observe a strong influence of the CDR3 loop states on the relative interface angle distributions.

**Figure 4:**
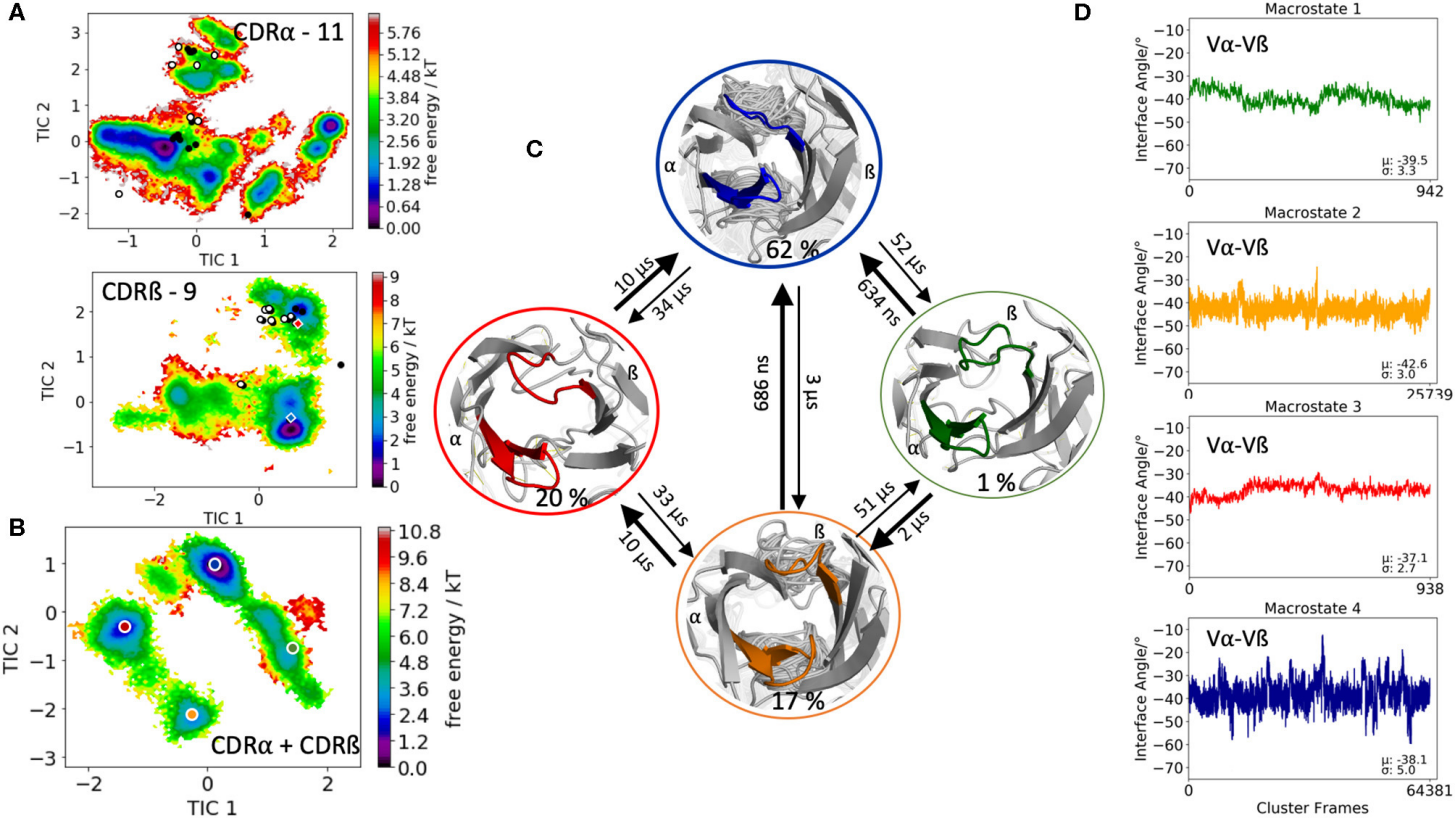
Free energy surfaces of the individual and combined CDR3 loops including a Markov-state model and the relative interdomain distributions upon conformational changes in the CDR3 loops of the E8 TCR. **(A)** Individual CDR3α and CDR3ß conformational spaces including the projections of the respective CDR3 loop crystal structures of same loop length. The respective CDR3 loop lengths are included in the individual free energy surfaces. The X-ray structures crystallized without antigen, are colored in white, while the complexed structures are colored black. Additionally, the crystal structures (diamond shapes) of the same TCR crystallized with (blue) and without (red) the pMHC complex are projected into the individual free energy landscapes. **(B)** Combined free energy surface of both CDR3 loops including the projections of the resulting four macrostate representatives. **(C)** Markov-state model, including the respective four macrostate ensembles and transition kinetics. The color-coding of the individual macrostates corresponds to the combined free energy surface in **(B)**. **(D)** Relative Vα-Vβ interdomain orientations for the individual macrostates reveal small shifts upon conformational changes in the CDR3 loops. Again, the color-coding corresponds to **(B,C)**.

The fourth TCR investigated, is the human melanoma specific TCR (E8) complexed with the MHC molecule and an epitope variant of the triosephosphate isomerase. E8 revealed very low affinity for the mutant triosephosphate isomerase-HLA-DR1, even though, the E8 cells have highly tumor-reactive properties (55). Three X-ray structures of the E8-TCR crystallized with and without antigen were available (PDB accession codes: 2IAM, 2IAN, 2IAL). As starting structure, we used the E8-TCR crystallized with antigen with the PDB accession code 2IAM. The biggest conformational differences upon antigen binding are located in the CDR3 loops. The CDR3α loop length is 11, while the CDR3ß loop consists of 9 amino acids. The clustering of 1 μs of metadynamics simulation resulted in 93 clusters and the obtained 9.3 μs of trajectories were used to reconstruct thermodynamics and kinetics of the CDR3 loop conformational rearrangements in solution. Again, the individual CDR3α and CDR3ß loop conformational spaces are illustrated in Figure 4A and we observe that again the majority of available TCR crystal structures are present in solution. The combined free energy surface of the CDR3 ensembles is illustrated in Figure 4B. The Markov-state model in Figure 4C resulted in four macrostates with transition kinetics in the nano-to-microsecond timescales. We further used the macrostate ensembles to calculate the relative interdomain orientations to identify shifts upon conformational changes in the CDR3 loops (Figure 4D). In this case, we only observe small shifts in the interdomain angle distributions for different CDR3 loop conformational states.

**Figure 5:**
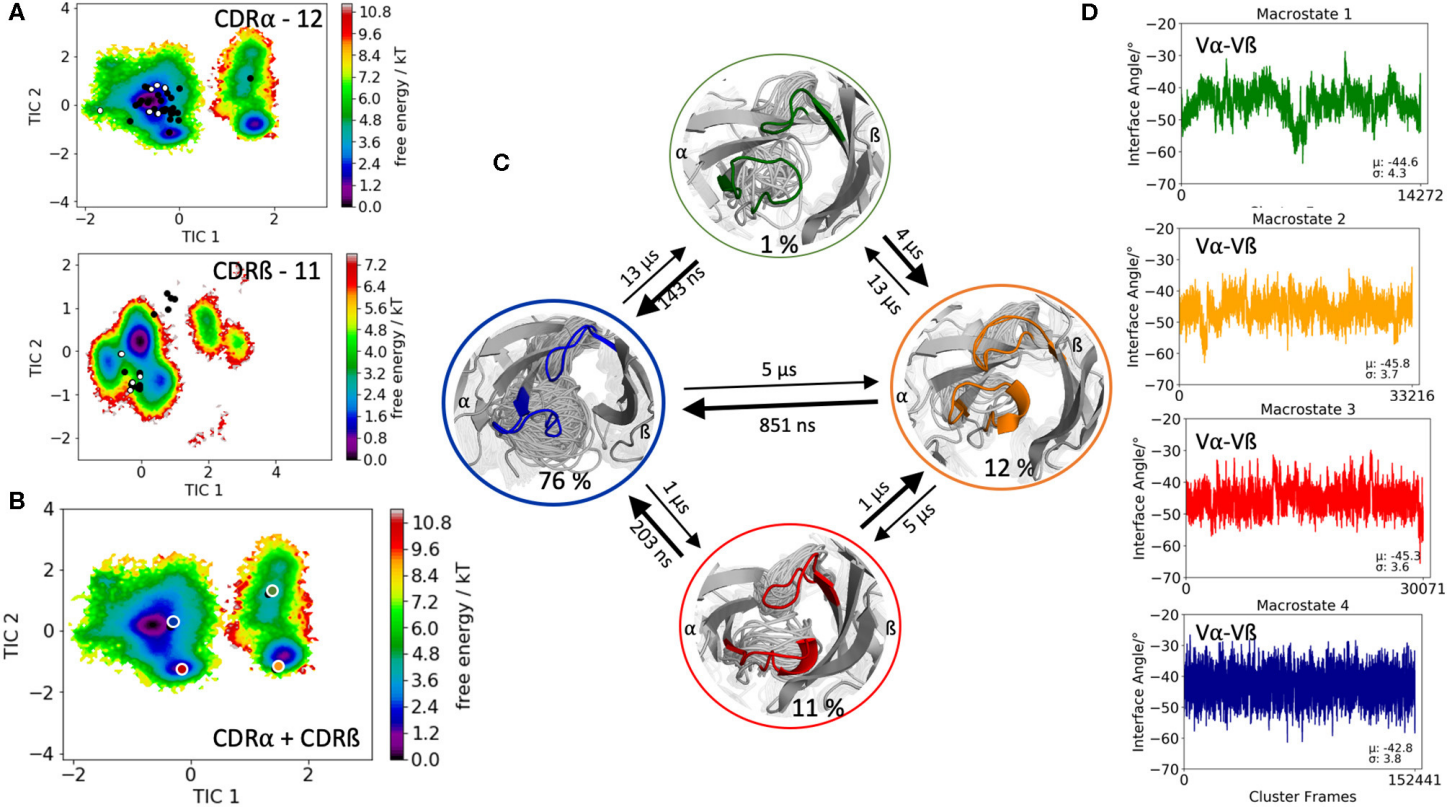
Free energy surfaces of the individual and combined CDR3 loops including a Markov-state model and the relative interdomain distributions upon conformational changes in the CDR3 loops of the 42F3 TCR. **(A)** Individual CDR3α and CDR3ß conformational spaces including the projections of the respective CDR3 loop crystal structures of same loop length. The respective CDR3 loop lengths are included in the individual free energy surfaces. Additionally, the crystal structures (diamond shapes) of the same TCR crystallized with (blue) and without (red) the pMHC complex are projected into the individual free energy landscapes. The X-ray structures crystallized without antigen, are colored in white, while the complexed structures are colored black. **(B)** Combined free energy surface of both CDR3 loops including the projections of the resulting four macrostate representatives. **(C)** Markov-state model, including the respective four macrostate ensembles and transition kinetics. The color-coding of the individual macrostates corresponds to the combined free energy surface in **(B)**. **(D)** Relative Vα-Vβ interdomain orientations for the individual macrostates reveal small shifts for conformational rearrangements in the CDR3 loops. Again, the color-coding corresponds to **(B,C)**.

The last TCR studied, is the 42F3 TCR, which is derived from an alloreactive cytotoxic T lymphocyte clone that recognizes MHC class I molecule H2-L^d^ presenting peptide p3A1 (PDB accession code 3TJH). The 42F3 TCR is related to another p-H2-L^d^ reactive TCR, the 2C TCR, which both recognize the related epitope of 2-oxogluterate dehydrogenase, QL9. The only differences between the two TCRs are the different CDR3 sequences to recognize QL9-H2-L^d^ (56). The CDR3α loop contains 12 residues, while the CDR3ß loop consists of 11 amino acids. Clustering resulted in 230 cluster representatives, which were used as starting structures for each 100 ns of molecular dynamics simulations. The 23.0 μs of trajectories were used to reconstruct kinetics and thermodynamics of the conformational rearrangements in the CDR3 loops and the influence of different CDR3 loop conformations on the relative Vα-Vβ distributions. Figure 5A illustrates the free energy landscapes of the individual CDR3α and CDR3ß loops including the projections of the available TCR CDR3 loop crystal structures of same length. Again, the majority of X-ray structures is present for the CDR3α loop even another dominant minimum in solution could be identified, which is not apparent from X-ray maybe due to crystal packing effects. The combined free energy surface of the CDR3 loops and the respective Markov-state model are displayed in Figures 5B,C. We obtained four macrostates with kinetics in the low microsecond timescale. Additionally, we observe small shifts in the Vα-Vβ distributions for different CDR3 loop macrostates upon conformational changes in the CDR3 loops. Figure S9 shows a summary table of all observed Vα-Vβ distributions with the respective averages and variances. To enhance the statistical significance of our results, we performed the Kolmogorov-Smirnov test to compare all macrostates of one TCR with each other (57). The respective *p*-values are illustrated in Figure S10. We clearly see that most of the distributions are different from each other and that conformational changes in the CDR3 loops, result in shifted interface angle distributions. Additionally, to show the CDR loop flexibilities, especially of the CDR3 loops, we included in Figure S11 the resulting RMSF plots of the respective macrostates.

## Discussion

The outstanding flexibility and dynamics of TCR CDR loops has been the focus of various crystallographic and computational studies (16, 19). The same TCR was shown to adopt multiple loop conformations, indicating their crucial role in cross-reactivity (58). Additionally, a characterization of structure and dynamics of a TCR is essential to elucidate the antigen-binding mechanism, the involved conformational changes and the associated biological implications (4, 17, 18). X-ray studies comparing TCRs crystallized with and without the pMHC complex revealed a high flexibility in the binding interface (1, 3, 14, 17, 20). However, crystal structures represent only a snapshot of a specific protein conformation that can be distorted due to crystal packing effects (15, 59). Studies on antibodies and TCRs discussed the high flexibility of the CDR3 loops and the fact that a single static structure is not sufficient to capture the high conformational diversity of these loops (15, 16, 60–62). It has also been shown that the CDR3α loop exhibits slower and simpler movements, while the CDR3ß loop displays faster and more diverse loop rearrangements (16, 63). Characterizing the conformational diversity of TCR binding loops has been shown to play a crucial role in elucidating the antigen-binding mechanism and the TCR binding specificity (18, 64, 65). The concept of conformational selection suggests, that within this pre-existing ensemble of conformations, the binding competent state is selected, accompanied by a population shift of the conformational states (66–68). Depending on the probability of the conformation chosen by the binding partner, the binding mechanism is called “lock and key,” “conformational selection,” or “induced fit.” If the dominant apo state is selected as binding competent conformation, the binding is normally denoted as “lock and key” (69). If the binding selects an apo conformation that is observed before binding, i.e., this conformation has a high enough probability to be within the arbitrary detection limit of an experimental technique, the binding is called “conformational selection” (67, 68). Finally, if the binding occurs to a rare conformation, that is present before binding, but cannot be detected before binding—all conformations pre-exist, however, with varying probabilities—the process is interpreted as “induced fit” binding (Figure 7) (70).

**Figure 6:**
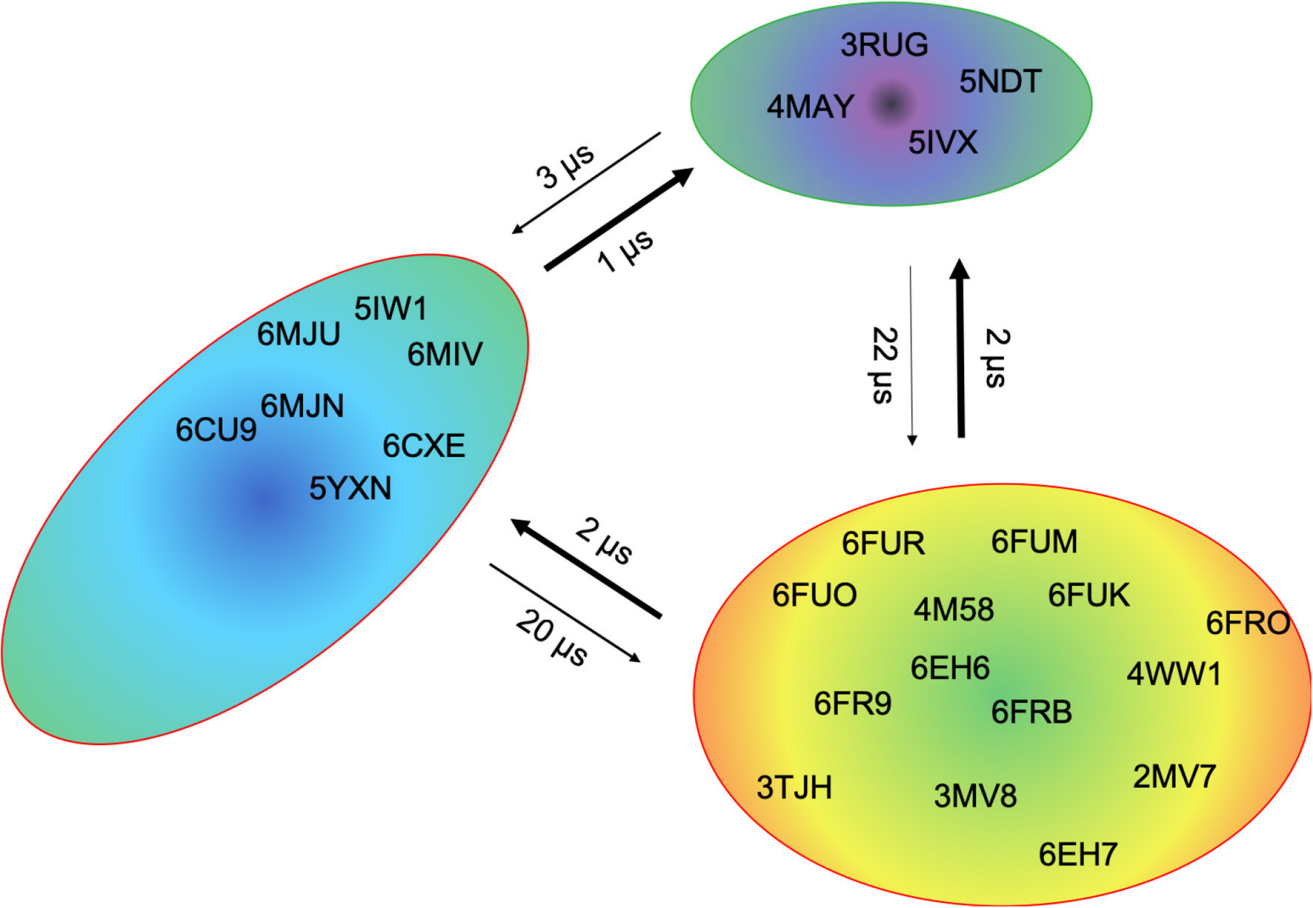
Assignment of the available TCR crystal structures to kinetic macrostates for the B4.2.3 TCR CDR3β loop with a loop length of 10 residues. The state probabilities correspond to the free energy surface in Figure 1A and are indicated in the color-coding. Additionally, transition timescales between the different macrostates in the low microsecond timescale can be observed.

This view on proteins that one sequence shows high conformational diversity and thus can adopt various conformations, facilitated the understanding of the evolution of new structures (71). A prime example for conformational selection is illustrated in Figure 1A. The B4.2.3 TCR binding to the pMHC ligand P18-I10 H2-D^d^ exhibits substantial structural differences upon binding in particular, in the CDR3 loops. Especially for the CDR3ß loop, we clearly see that even without the presence of the antigen the binding competent conformation lies in the dominant minimum in solution, while the X-ray structure crystallized without the antigen is situated in a local side minimum (Figure 1A). These observed structural changes in the CDR3 loops can be explained due to interactions of the TCR CDR3 loops with the tail region of the crystal symmetry mates (15, 72). Additionally, Figure 1A illustrates the high mobility and flexibility of the CDR3 loops and thereby provides insights into the immune recognition and selection mechanisms. The majority of available crystal structures are present within our obtained ensembles in solution. A possible assignment of the crystal structures to kinetic macrostates in solution is shown in Figure 6 for the CDR3ß loop. Various studies focused on characterizing the role of the CDR3 loops and identified strong structural loop correlations in the peptide recognition process (13, 14, 19). As evidence for a structural correlation of the CDR3 loops has been provided, we reconstructed thermodynamics and kinetics for the combined CDR3 loops. The combined CDR3 conformational space in Figure 1B displays a shallow free energy surface with five macrostates. Excitingly, we were able to observe substantial shifts in the relative Vα-Vβ distributions for conformational rearrangements in the CDR3 loops, indicating a strong dependency of the CDR3 loop conformations on the relative interface angle orientations. This strong influence of different CDR3 loop conformations can be explained because of their location in the center of the paratope and their major role in recognizing and binding to the peptide. In line with these results shifts in the relative Vα-Vβ distributions can be observed for all studied TCRs (Figures 2D, 3D, 4D, 5D) highlighting the extraordinary conformational diversity and the correlation between the CDR3 loops and the interface angles. Previous studies have shown that the relative interdomain orientations reveal a high variability and fluctuate in the 0.1 GHz timescale. These results suggest that short molecular dynamics simulations are sufficient to capture the majority of accessible interdomain orientations (73). In agreement with these studies we observe that the slow component of the relative Vα-Vβ dynamics strongly correlates with conformational CDR3 loop rearrangements, which occur on the micro-to-millisecond timescale.

**Figure 7:**
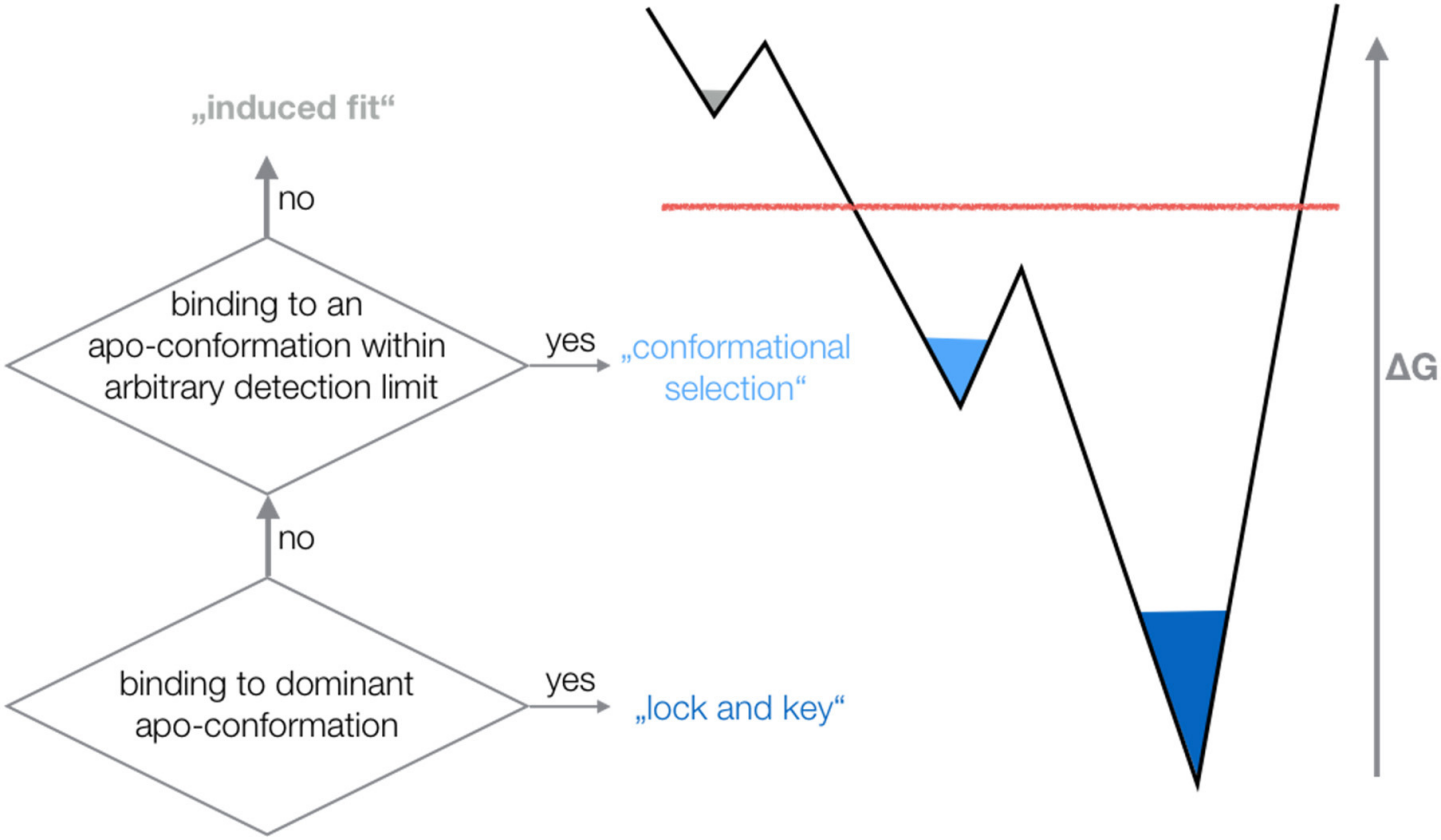
Schematic illustration of different binding mechanisms and their relationship to conformational ensembles before binding. Depending on the probability of the conformation chosen by the binding partner, the binding mechanism is called “lock and key,” “conformational selection,” or “induced fit”.

Another example of conformational selection is illustrated in Figure 4A. In line with the results in Figure 1A, even without the presence of the antigen the binding competent conformations of both the CDR3α and CDR3ß are present and lie in the global minimum in solution, while the X-ray structure crystallized without antigen is distorted due to crystal packing effects and is located in a side minimum. Figure S6 shows both the CDR3α and CDR3ß conformational spaces, resulting of 14.4 μs of trajectories, by using the X-ray structure, crystallized without antigen (PDB accession code 2IAL) as starting structure for molecular dynamics simulations. Also, in this example we observe, especially for the CDR3α, in agreement with the results shown in Figure 4A, that the binding competent state is the dominant structure in solution, while the apo structure lies in a local shallow side minimum. Figures S7, S8 show the observed crystal packing effects of the TCRs (Figures 1, 4, respectively) with their symmetry mate TCRs, highlighting the close contacts and interactions. These interactions and contacts result in different CDR3 loop conformations, which lie in local shallow side minima, while the complexed structures are present in the dominant minimum in solution. This distortion of CDR loops has already been observed and discussed in various examples for antibody Fabs, showing that antibodies are optimized to bind the antigen (15, 72). The fact, that the binding competent CDR3 loop conformations lie in the global minimum in solution, strengthened the confidence in our results. The 003 TCR recognizing HIV p17 Gag-derived peptide presented by HLA-A^*^0201 has already been studied to investigate the TCR loop flexibility and the influence of crystallization conditions on the resulting crystal structures. Our results are in perfect agreement with the observations of this study, that one single snapshot of a TCR is not enough to capture and understand this high conformational diversity. Especially the CDR3ß conformational space in Figure 3B illustrates the high flexibility of this crucial binding loop for peptide recognition, because not only the majority of available TCR crystal structures are present within the resulting ensemble in solution, but also another minimum could be identified, which is not apparent from X-ray structures, maybe due to crystal packing effects. Analogous to these observations, Figure 2A shows for both the CDR3α and CDR3ß loops that besides capturing the majority of crystal structures, additional dominant solution structures have to be considered when characterizing CDR3 loops.

## Conclusion

In this study we investigated five T-cell receptors with different CDR3 loop lengths and identified that the majority of available crystal structures of the same CDR3 loop lengths are present within the CDR3 loop ensembles in solution and some of them even belong to the same kinetic minimum. Thus, we observe that one TCR CDR3 loop can adopt various conformations and thereby clearly follows the concept of conformational diversity. Additionally, these findings extend the model of static canonical clusters to a dynamic conformational ensemble. Thereby, to properly capture this high flexibility, the CDR3 loops need to be characterized as conformational ensembles. To identify the influence of the CDR3 loop conformational states on the relative Vα-Vβ distributions we calculated a combined Markov-state model and the respective CDR3 macrostates in solution exhibited strong shifts in the relative interdomain Vα-Vβ distributions for all studied TCRs upon changes in the CDR3 loop conformations.

## Data Availability Statement

All datasets presented in this study are included in the article/Supplementary Material.

## Author Contributions

The manuscript was discussed and written through contributions of all authors. All authors have given approval to the final version of the manuscript.

## Conflict of Interest

The authors declare that the research was conducted in the absence of any commercial or financial relationships that could be construed as a potential conflict of interest.
