# Supplementary material for: T-Cell Receptor CDR3 Loop Conformations in Solution Shift the Relative Vα-Vβ Domain Distributions
Source: Front Immunol. 2020 Jul 8;11:1440. doi: 10.3389/fimmu.2020.01440 (PMC7360859; doi:10.3389/fimmu.2020.01440)
Supplement: Supplementary file 1 [file Data_Sheet_1.docx]

#
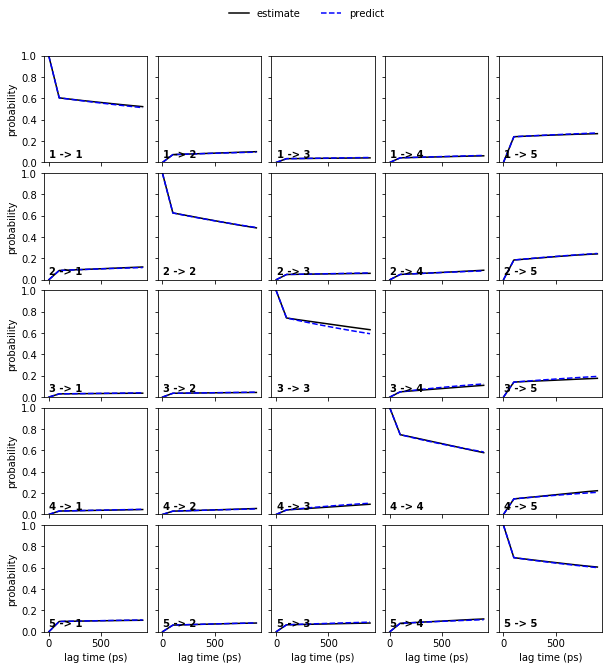


SI Figure S1: Markov-state model validation tool. Chapman-Kolmogorov tests for the Markov-state models of the B4.2.3 TCR.


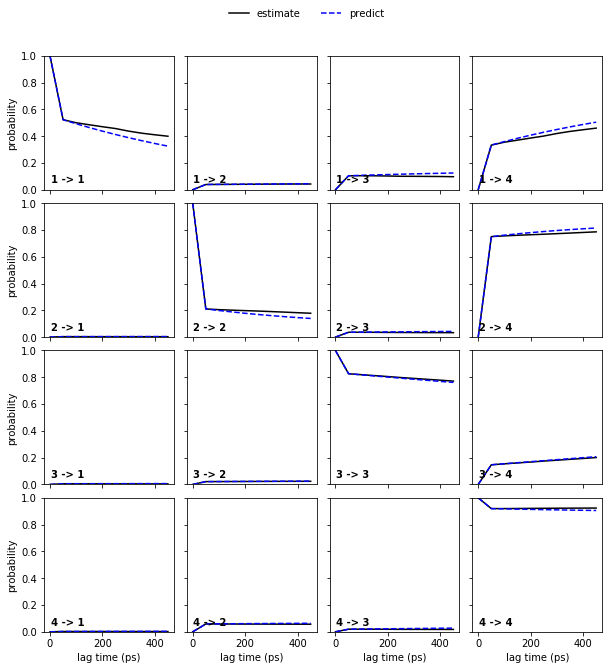


SI Figure S2: Markov-state model validation tool. Chapman-Kolmogorov tests for the Markov-state models of the B4.2.3 TCR.


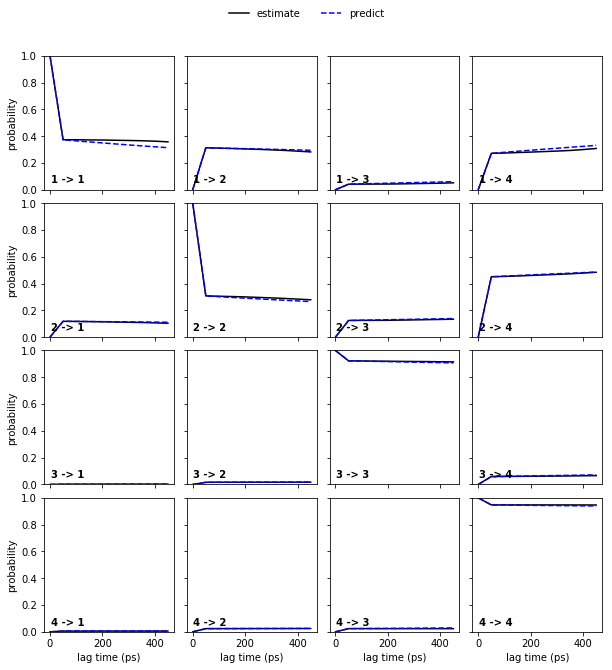


SI Figure S3: Markov-state model validation tool. Chapman-Kolmogorov tests for the Markov-state models of the 003 TCR.


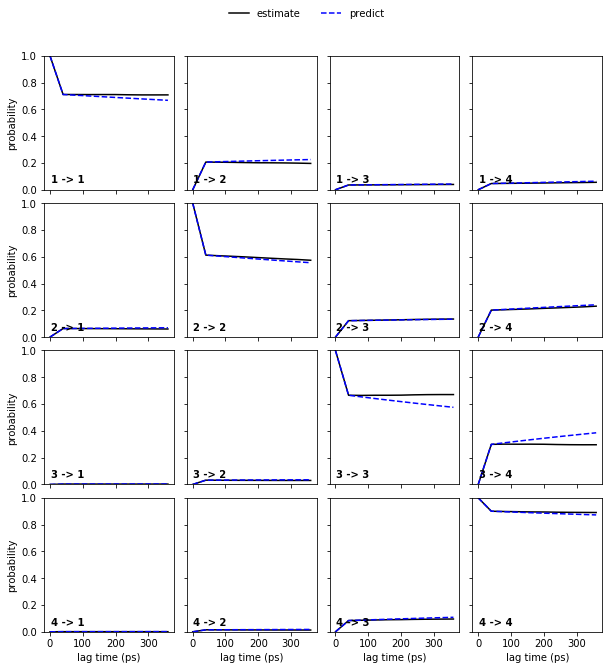


SI Figure S4: Markov-state model validation tool. Chapman-Kolmogorov tests for the Markov-state models of the E8 TCR.


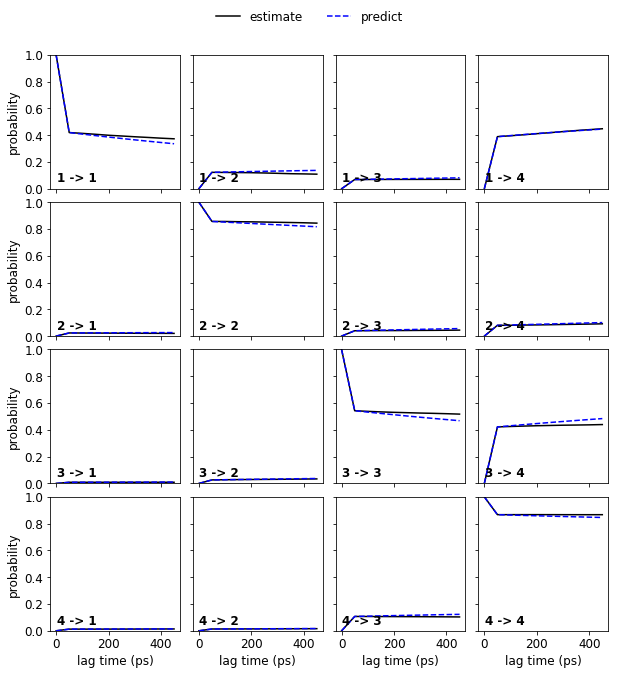


SI Figure S5: Markov-state model validation tool. Chapman-Kolmogorov tests for the Markov-state models of the 003 TCR.


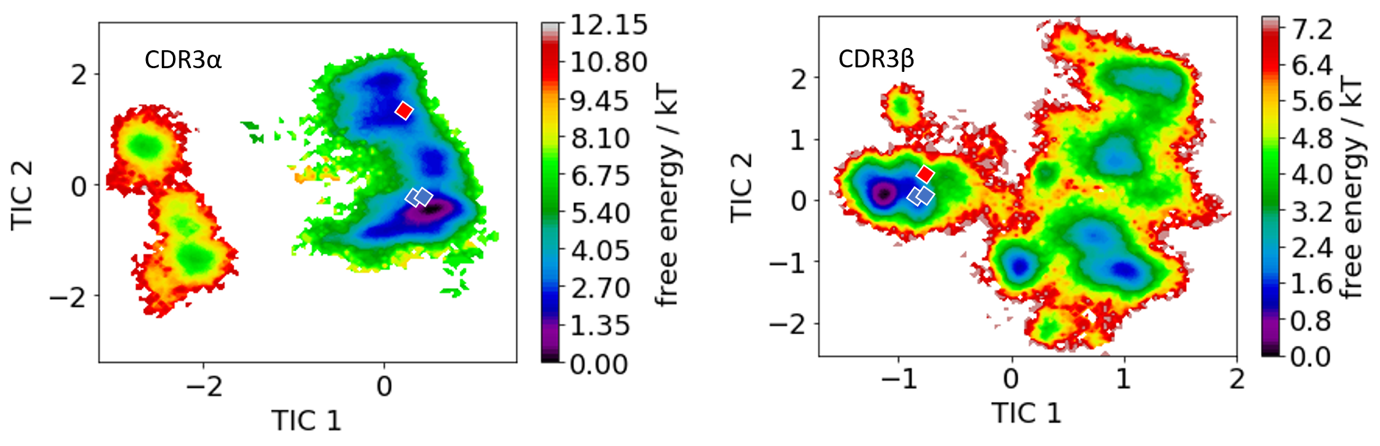


SI Figure S6: Free energy surfaces of the CDR3α and CDR3β loops of the E8 TCR antibody starting from the X-ray structure crystallized without the antigen. The blue diamonds represent the complexed X-ray structure, while the red diamond represents the apo crystal structure (PDB accession code 2IAL).


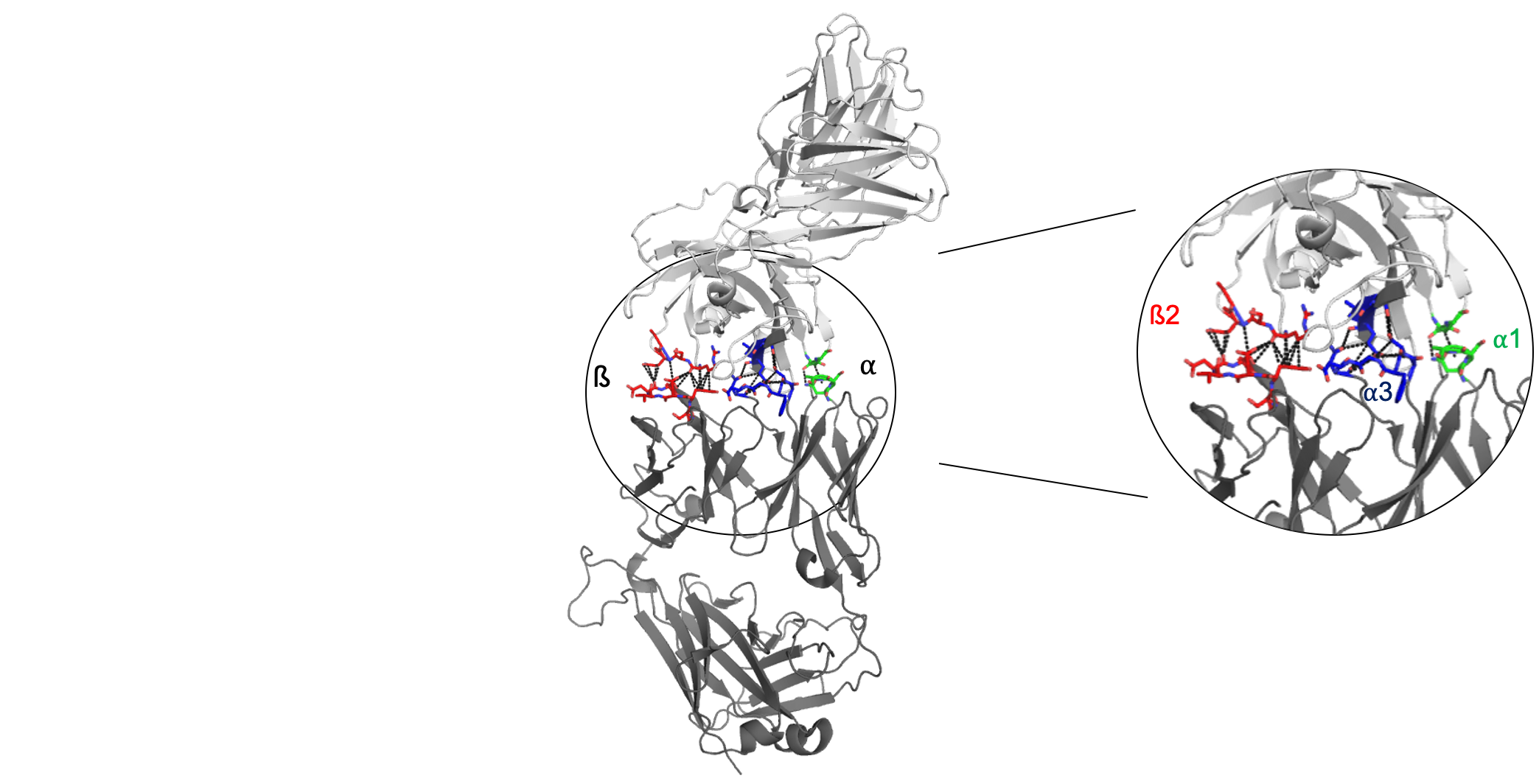


SI Figure S7: Crystal contacts of the apo TCR (5IW1) with the tail of a symmetry mate (light grey), which causes a rearrangement of the CDR3 loops. The residues showing interactions with the CDR3 loops are colored and labeled respectively.


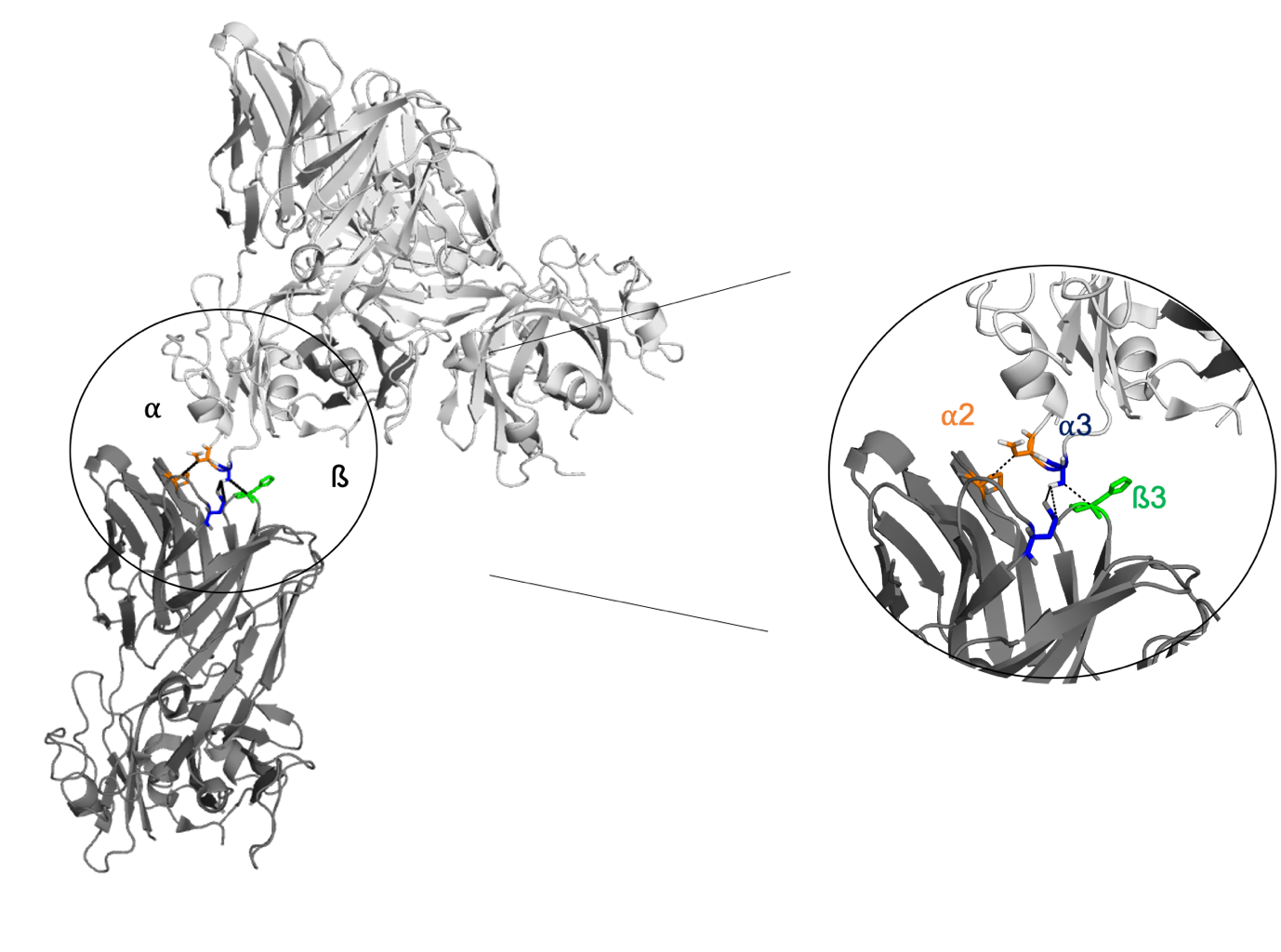


Figure 8: Crystal contacts of the apo TCR (2IAL) with the tail of a symmetry mate (light grey), which causes a rearrangement of the CDR3 loops. The residues showing interactions with the CDR3 loops are colored and labeled respectively.


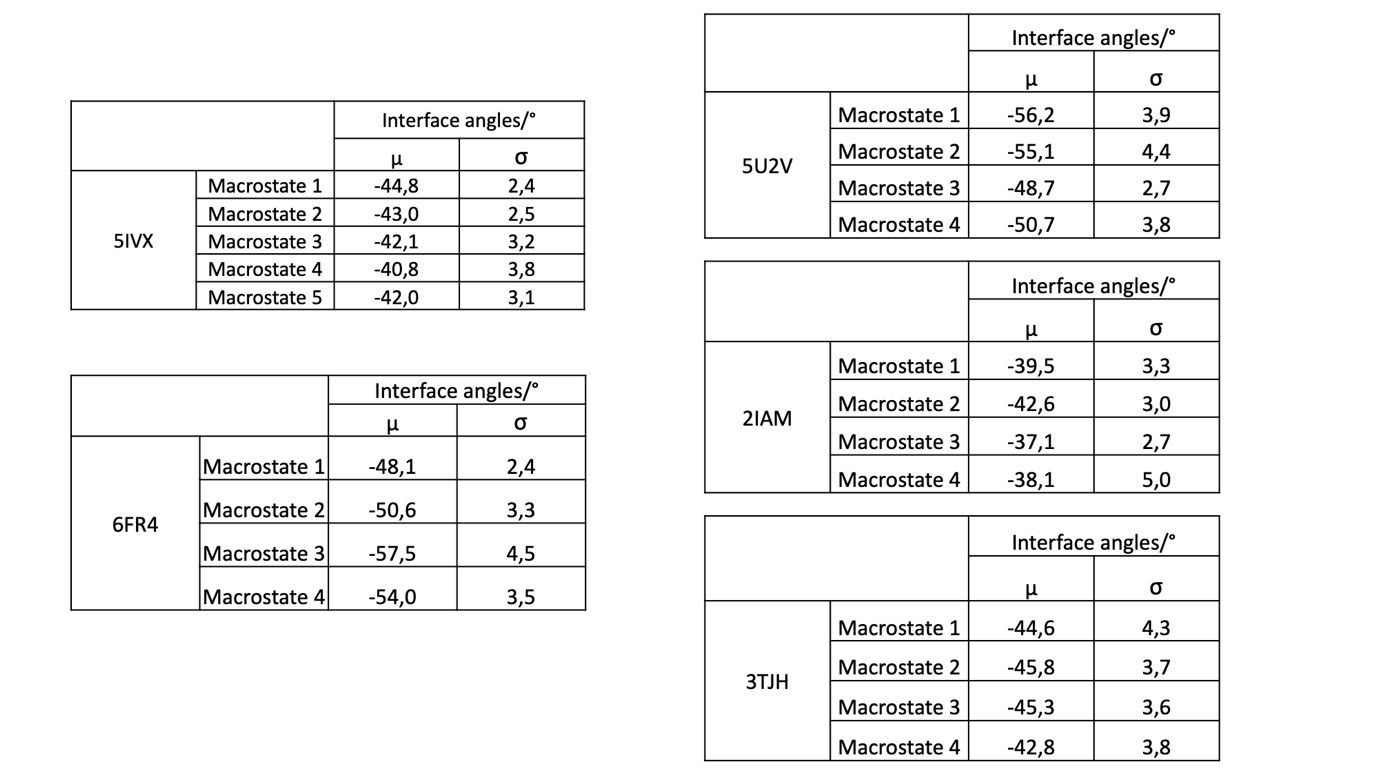


SI Figure S9: Summary of all interface angles including the average and the respective variance.


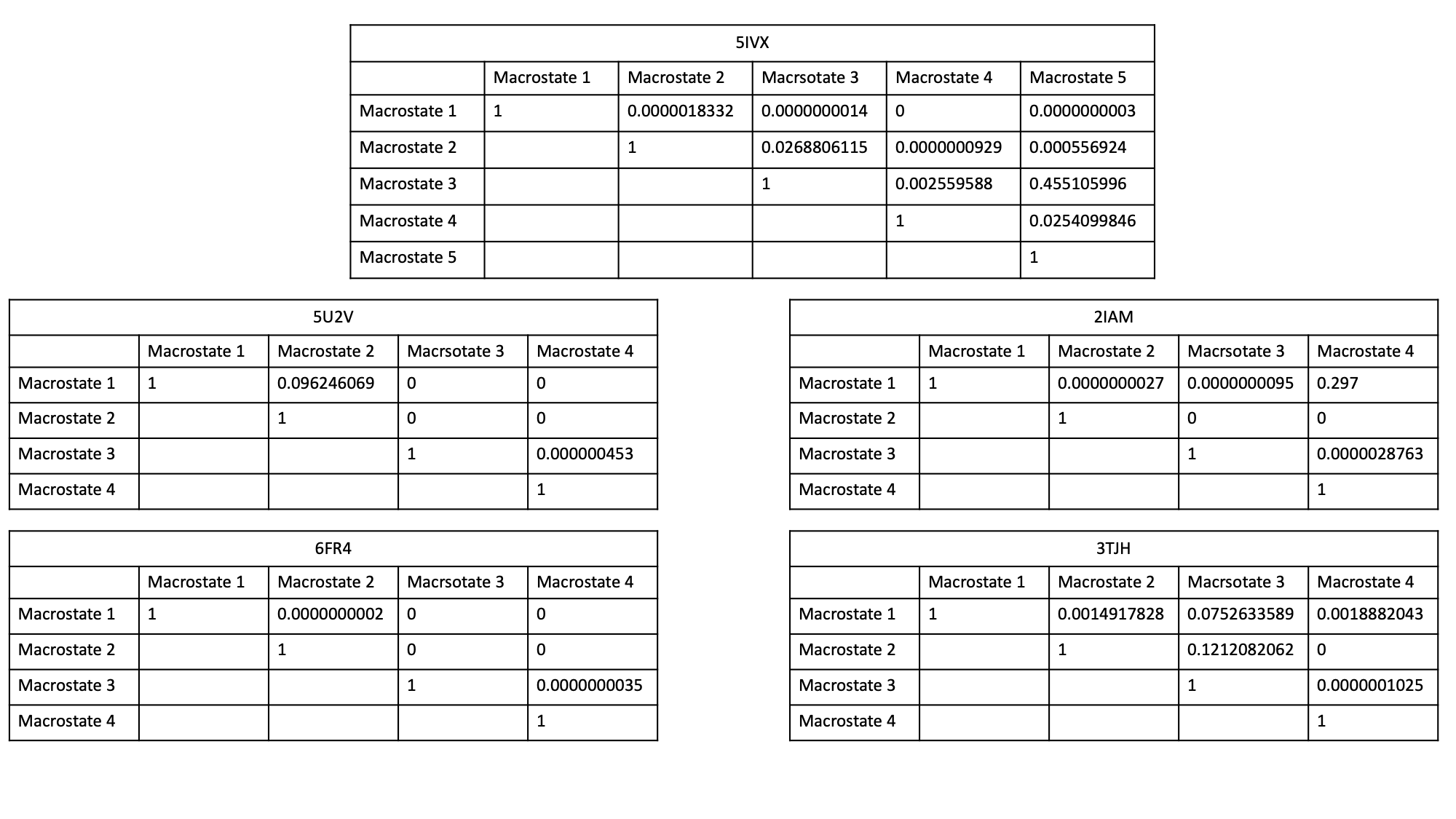


SI Figure S10: p-values indicating the significance of the shifts in the relative V_H_-V_L_ distributions by using the Kolmogorov-Smirnov test for all studied TCRs.


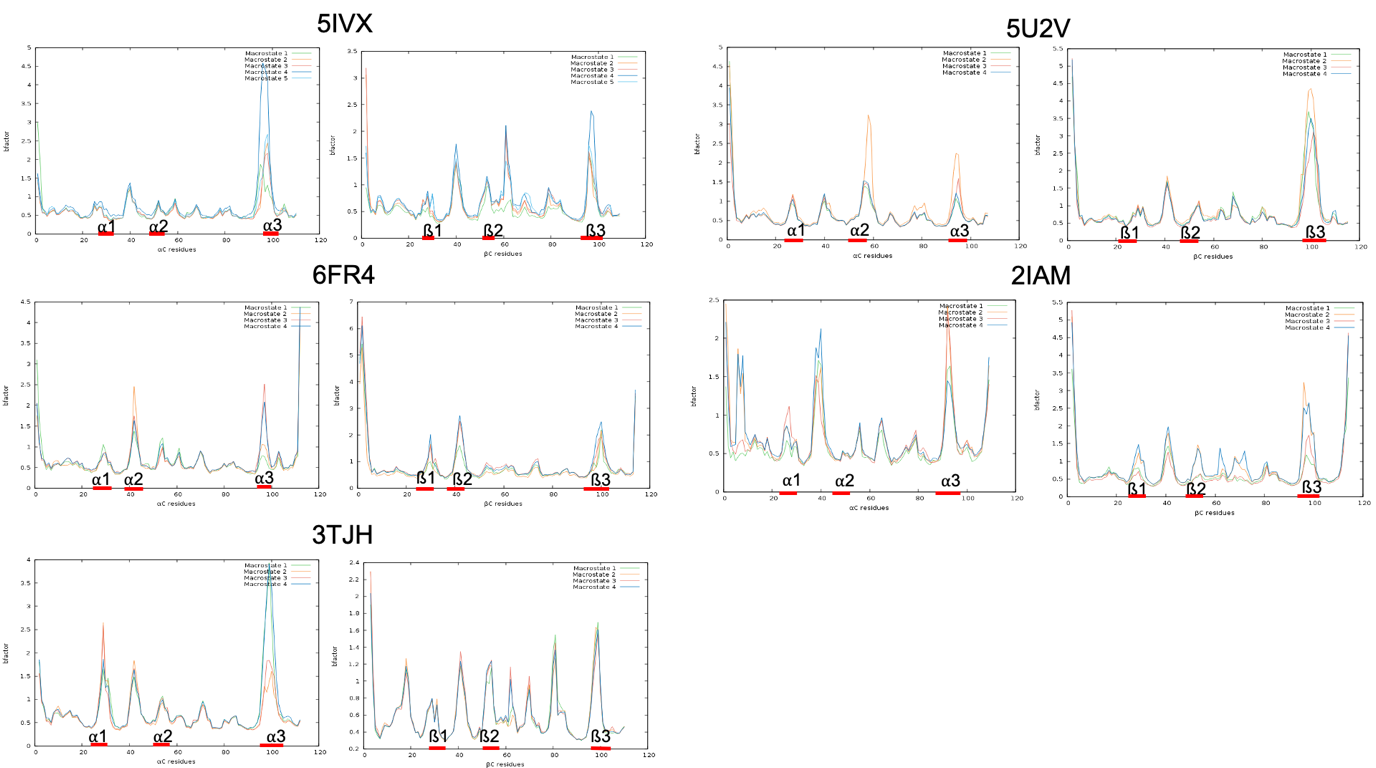


SI Figure S11: RMSF plots of the individual macrostates for all systems, showing the high flexibility of the CDR3 loops. However, in some cases also the loops pointing towards the constant domains reveal a high flexibility.
